# Whole-Genome DNA Methylation Analysis of Inoculation with *Trichothecium roseum* in Harvested Muskmelons

**DOI:** 10.3390/jof11040243

**Published:** 2025-03-22

**Authors:** Liang Lyu, Lei Li, Chenglong Zhao, Yuchao Ning, Yawen Luo, Xining He, Mina Nan

**Affiliations:** 1 College of Biological and Pharmaceutical Engineering, Lanzhou Jiaotong University, Lanzhou 730070, China; 2 Laboratory and Practice Base Management Center, Gansu Agricultural University, Lanzhou 730070, China

**Keywords:** DNA methylation, biotic stress, muskmelons, defense response

## Abstract

DNA methylation is a crucial epigenetic marker linked to plant defense responses, but its significance in fungal infection of postharvest fruits remains poorly understood. This study indicated that *Trichothecium roseum* inoculation increased ROS production, enhanced phenylpropanoid metabolism-related enzyme activity, and promoted lignin accumulation in harvested muskmelon fruits *(Cucumis melo* cv. Yujinxiang) within 24 h post-inoculation (hpi). In addition, whole-genome bisulfite sequencing showed that genomic DNA methylation levels of muskmelon decreased by 6.15% at 24 hpi. Notably, CG sites exhibited a higher methylation level and the largest number of differentially methylated regions (DMRs). Moreover, 176 DMR-associated genes (DMGs) involved in the defense response, 134 DMGs in the ROS metabolic pathway, and 41 DMGs in phenylpropanoid metabolism were identified. The differentially expressed genes harboring differential methylation were mainly influenced by hypomethylation and exhibited elevated transcript levels, involved in phenylpropanoid biosynthesis and biosynthesis of secondary metabolites.

## 1. Introduction

The epigenome is made up of DNA methylation, chromatin alterations, and histone modifications. The stability of chromatin and gene expression is significantly influenced by dynamic epigenetic mechanisms [1]. Differential DNA methylation in response to pathogen infection or unfavorable environmental conditions allows defense-related genes to be expressed selectively. Stress-related DNA hypomethylation and hypermethylation can trigger the defensive response in plants [1]. Plants’ epigenomes change during growth, development, and hybridization according to genome-wide DNA methylation studies [2,3], disease response [4,5], drought [6], and induced and spontaneous mutations [7,8,9]. Many studies have revealed patterns of DNA methylation across diverse plant genomes, including *Arabidopsis* [10], rice [7], wheat [11], maize [12], *Brassica juncea* [6], and *Plantago lagopus* [13], which indicate their reliance on transcription processes. DNA methylation in several plants has been successfully profiled using whole-genome bisulfite sequencing (WGBS), which has shown its function in regulating male sterility in *Brassica napus* [14], regulating daily gene expression in *Populus trichocarpa* [15], and responding to *Cucumber green mottle mosaic virus* infection in watermelons [5]. Applying WGBS to study DNA methylation dynamics in fruits during pathogen infection will help to explore the associated host response mechanisms.

The muskmelon (*Cucumis melo* L., 2n = 2x = 24) is a crop with significant global importance because of its extensive production and consumption [16]. However, fungal infections cause a 20–40% loss after harvest [16]. *Trichothecium roseum*, an influential fungus that affects crops after harvest, is known for leading to pink rot in muskmelons grown in the northwestern part of China, the nation’s largest production zone [17]. The plants of muskmelon have developed sophisticated immune systems that control the metabolism of reactive oxygen species in order to identify pathogen infections and trigger subsequent defense mechanisms [18], phenylpropanoid metabolism [19], and energy metabolism [20,21]. In this study, we examined the early defense mechanisms in fungus-infected muskmelon fruits, with a particular emphasis on the build-up of reactive oxygen species and modifications in important phenylpropanoid pathway enzymes. In addition, we employed WGBS to examine DNA methylation patterns in muskmelon, and comparative transcriptomic and methylomic analyses were performed to identify differentially expressed genes (DEGs) and genes associated with differentially methylated regions (DMGs) in response to *T. roseum* infection. These results shed light on the potential roles of DNA methylation in muskmelon fruits during the early defense stage.

## 2. Materials and Methods

### 2.1. Fruit Material and Trichothecium roseum Inoculation

Muskmelons (*Cucumis melo* L. cv. Yujinxiang) were harvested in Lanzhou City, located in Northwest China. The fruits were harvested at the start of physiological maturity (35 days after full blossom), and the fruits were selected for uniformity of size and shape, without infection, diseases, or physical injuries. The fruits were packed in cartons and transported to the laboratory at Gansu Agricultural University within six hours, then stored at room temperature (22 ± 2 °C, relative humidity 55–60%) for use. *Trichothecium roseum* (Pers.: Fr.) Link was isolated from infected melons, identified based on a conidial morphology and conidial attachment to conidiophores (erect, unbranched, aseptate, rarely with few septa), maintained on potato dextrose agar (PDA) at 4 °C. The conidia of the pathogens were obtained from 10-day-old PDA cultures and incubated at 25 °C for artificial inoculation (pathogen challenged—PC) [22]. For each group, 18 melons were used (CK, control check; PC, pathogen challenge), as well as three independent biological replicates, each comprising six melon fruits. All biochemical analyses were conducted in triplicate to ensure methodological rigor. The procedure for sample collection followed the guidelines laid out by Ren et al. [23].

### 2.2. Assay and Localization for ROS Production

The production of O_2_^•−^ followed the method described by Elstner and Heupel [24]. The concentration of H_2_O_2_ in the muskmelon fruits that were inoculated with fungi, as well as in the control samples, was measured following the procedure described by Ferguson et al. [25]. At 24 h post-inoculation (hpi), the levels of intracellular reactive oxygen species (ROS) were assessed using a 10 mM oxidant-sensitive probe, 2′, 7′-dichlorofluorescein diacetate (DCHF-DAh), following the protocol developed by Lyu et al. [21].

### 2.3. Assays for Enzyme Activity and Lignin Content

PAL activity was assayed as described by Liu et al. with some modifications [19]. The reaction solution contained 3 mL of L-phenylalanine for PAL and 500 μL of crude enzyme extract. The mixture was incubated at 40 °C for 1 h and then halted by adding 0.2 mL of 6 M HCl. Absorbance at 290 nm was measured for PAL, and the PAL activity was reported as 0.01 ΔOD_290_/mg protein.

C4H activity was determined according to the method described by Lamb and Rubery, with some modifications [26]. The reaction mixture contained 2 mL of 50 mM Tris–HCl buffer (pH 8.9; containing 8 μM trans-cinnamic acid, 3 μM NADPNa_2_, 6 μM G-6-PNa_2_) and 0.2 mL of crude enzyme. After blending, the mixture was incubated at 25 °C for 30 min, and the reaction was terminated by adding 100 μL of 6 M HCl. C4H activity was expressed as 0.01 ΔOD_340_/mg protein.

4CL activity was assessed using the method described by Voo et al. with some modifications [27]. A total of 0.5 mL of crude enzyme was combined with a reaction mixture composed of 0.45 mL of 75 mM MgCl_2_, 0.15 mL of 1 μM coenzyme A (CoA), 0.15 mL of 0.8 mM adenosine triphosphate, and 0.15 mL of 2 mM p-cumaric acid. The absorbance at 333 nm was recorded, and 4CL activity was quantified as 0.01 ΔOD_333_/mg protein.

CAD activity was measured according to Goffner et al. with some modifications [28]. The reaction mixture consisted of 0.6 mL of crude enzyme, 1 mL of 2 mM NADP, and 1.4 mL of 1 mM trans-cinnamic acid. After incubation at 37 °C for 30 min, 200 μL of 1 M HCl was added to halt the reaction. CAD activity was quantified as 0.01 ΔOD_340_/mg protein.

POD activity was assayed colorimetrically with guaiacol as the hydrogen donor according to the method of Venisse et al. with some modifications [29]. The POD activity was expressed as U/mg protein, where U = 0.01 ΔOD_470_/min. 

Determination of lignin content was carried out following the modified protocol of Liu et al. [19]. The lignin content was represented as ΔOD_280_ per gram of fresh weight (FW). According to Bradford [30], the protein concentration in the supernatants was analyzed.

### 2.4. Construction of Methyl-Seq Libraries

Genomic DNA from muskmelon was extracted using the E.Z.N.A.^®^ Tissue DNA Kit (Omega Bio-tek, Norcross, GA, USA) as per the manufacturer’s instructions. Only high-quality DNA samples with OD 260/280 ratios between 1.8 and 2.0 and a quantity exceeding 6 µg were utilized for constructing the fragment library. The method described by Bhat et al. [4] and Sun et al. [5] was used for the construction of the methyl SEQ libraries. Three biological replicates per sample were used. Shanghai OE Biotech Co., Ltd. in Shanghai, China performed WGBS. Six DNA samples, with three individuals per group, were subjected to WGBS. The library preparation followed the OE Biotech standard directional WGBS pipeline, involving sonication to fragment the genome into 100–300 bp fragments. Bisulfite treatment was carried out using the EZ DNA methylation Gold Kit (ZYMO, Mexico City, Mexico). WGBS was performed as 150 bp paired-end sequencing with a genome coverage of 12× using a NovaSeq 6000 system (Illumina, Inc., San Diego, CA, USA).

### 2.5. Data Processing

Fastp v0.20.0 was used for preprocessing raw reads, offering low-complexity filtering, polyX trimming at the 3′ ends, and base correction in overlapping regions [31]. Clean BS-Seq reads were aligned to the *Cucumis melo* L. cv. DHL92 reference genome of muskmelon. The differential methylation analysis was then carried out as follows using Methylkit (version 1.28.0) [32] in R ggseqlogo (version 0.1.1) [33]. Using the R program cluster Profiler (version 4.10.0), gene ontology enrichment analysis was performed, and important GO categories were chosen based on a q-value < 0.05 [34]. Using Sun et al.’s proposed method, the methylation level of each cytosine was determined by calculating the fraction of reads that showed mC among all reads covering the same cytosine [5].

### 2.6. RNA Sequencing and Data Analysis

RNA sequencing (RNA-seq) was performed on muskmelon fruits collected at 24 hpi, matching the stages of WGBS. The total RNA was extracted using TRIzol reagent (Invitrogen, Carlsbad, CA, USA) following the protocol provided by the manufacturer. After the total RNA was extracted, the mRNA was enriched using Oligo(dT) beads. Then, the enriched mRNA was fragmented into short fragments using fragmentation buffer and reverse transcribed into cDNA with random primers. These cDNA libraries were sequenced using the Illumina platform by Genedenovo Biotechnology Co., Ltd. (Guangzhou, China). The investigation of the connection between DNA methylation and gene expression adhered to the methodology described by Xu et al. [35].

### 2.7. Statistics Analysis

All analyses were conducted using the statistical software SPSS 17.0 (SPSS, Inc., Chicago, IL, USA). The data are presented as mean values ± standard errors (SEs) following the application of Duncan’s multiple range tests within a one-way analysis of variance (ANOVA).

## 3. Results

### 3.1. Fungal Infection Promoted ROS Accumulation in Muskmelon Fruits

**Figure 1:**
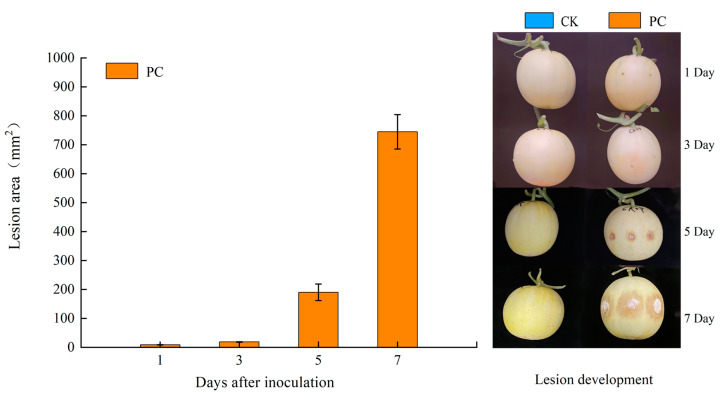
Lesion area development due to inoculation with *T. roseum* (PC, pathogen challenge; CK, control).

**Figure 2:**
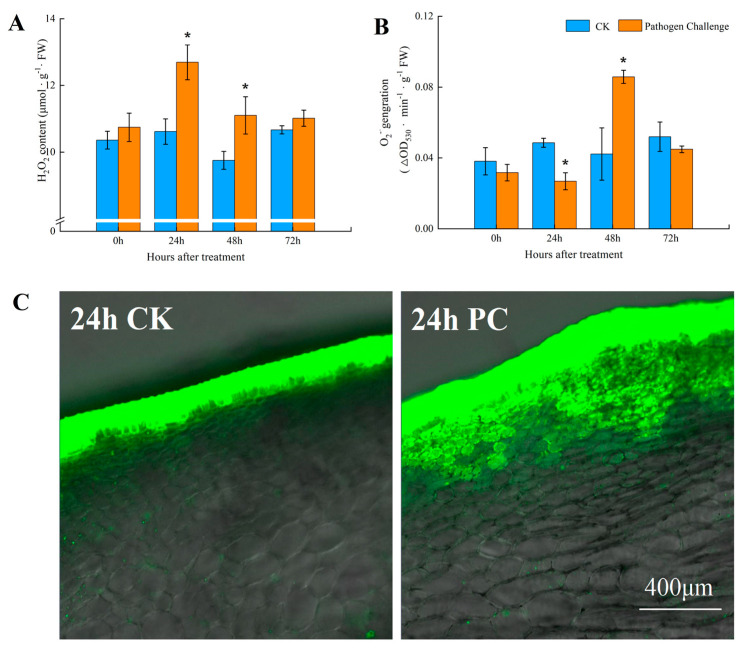
H_2_O_2_ content (**A**) and O_2_^•−^ generation rate (**B**) were measured in muskmelon fruits. (**C**) ROS detection near the inoculation point of tissue of muskmelon using the oxidant-sensitive probe 29,79-dichlorodihydrofluorescein diacetate (DCHF-DA). Scale bar, 400 μm; CK, control check; PC, pathogen challenge in early defense responses of muskmelon fruits after 24 h of inoculation. Asterisks indicate significant difference (*p* < 0.05).

During the first 3 d of inoculation, no discernible symptoms were found in the contaminated fruits (PC) and the controls (CK) (Figure 1). However, distinctive pink rot showed in the *T. roseum*-inoculated muskmelons 5 d after inoculation (Figure 2A).

The inoculation increased H_2_O_2_ concentration in the tissues at the junction of decay and healthy regions, and the value peaked at 24 hpi, which increased by 19.60% compared to the CK (*p* < 0.05) (Figure 2A). Additionally, the peak of O_2_^•−^ production was observed at 48 hpi, showing a 103.19% increase compared to the CK (*p* < 0.05) (Figure 2B). Moreover, a noticeable increase in the buildup of cellular ROS showed in the inoculated fruits. In particular, the infected hosts had more subdermal parenchyma cells stained with DCHF-DA than the CK at 24 hpi (Figure 2C). The accumulation of ROS and the fluorescent signals indicated significant ROS production with high fluorescence within the first 1 cm near the infection site in the early phase of the defense reaction.

### 3.2. The Activities of the Key Enzymes of the Phenylpropanoid Pathway After Fungal Infection

**Figure 3:**
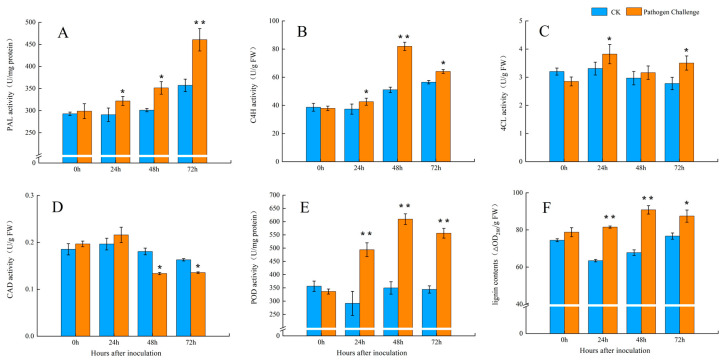
Changes in activity of PAL (**A**), C4H (**B**), 4CL (**C**), CAD (**D**), POD (**E**), and lignin contents (**F**) of muskmelon fruit challenged with *T. roseum* after 72 h; CK, control check; PC, pathogen challenge. Asterisks indicate significant differences, where * represents *p* < 0.05 and ** represents *p* < 0.01.

The PAL activity was lower in the CK compared with the inoculated group during incubation. The PAL activity increased gradually (*p* < 0.05) during the experiment, with the exception of the first two days following fungal infection (Figure 3A). The C4H activity showed an increase starting at 24 h, then peaked at 48 hpi (Figure 3B). Both 4CL and CAD activity increased at 24 hpi (Figure 3C,D), with subsequent decreases in activity, particularly in CAD. Fungal infection notably increased POD activity by 69.29% compared to the CK (Figure 3E, *p* < 0.05), with the highest POD activity observed at 48 hpi. Lignin content was significantly induced at 24 and 48 hpi, showing increases of 28.54% and 35.44%, respectively, compared to the CK (Figure 3F).

### 3.3. DNA Methylation Profiling

**Figure 4:**
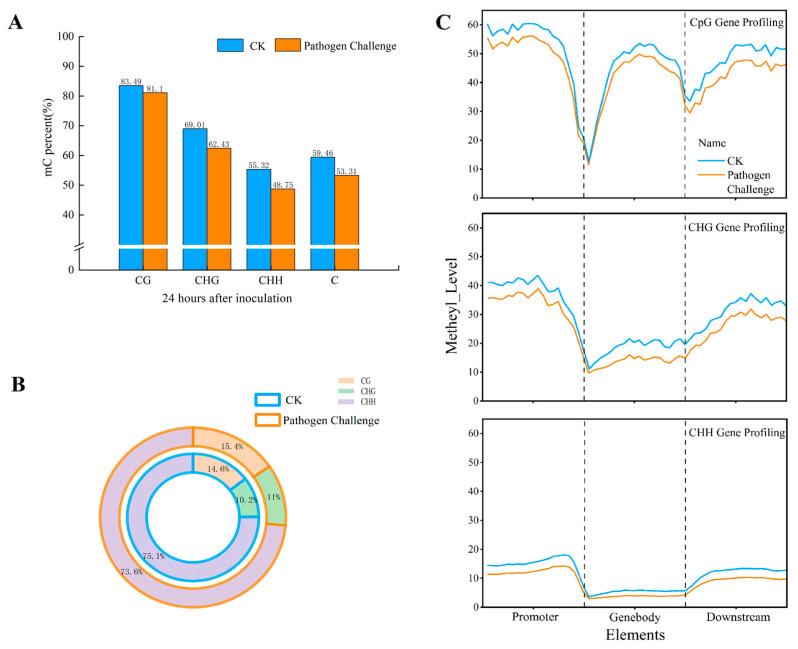
DNA methylation levels in muskmelon fruit challenged with *T. roseum* by WGBS at 24 hpi. (**A**) Global methylation pattern. (**B**) The proportion of each methylation context was compared between control (CK) and pathogen-challenged (PC) samples. (**C**) Changes in methylation levels were observed in the gene body, promoter, and downstream regions in the CG, CHG, and CHH contexts, respectively.

To unveil whether DNA methylation responded to *T. roseum* infection, methylome libraries were constructed with genomic DNA from the control and *T. roseum*-infected melon fruits. Control and pathogen challenged muskmelon fruits collected at 24 hpi were used for WGBS; a total of 139,163,300 and 138,433,467 clean reads were generated. The average read depth for the control and pathogen-challenged muskmelon fruits ranged from 20.70 G to 21.88 G, with mapping rates of 58.34% to 61.49%. In the control and pathogen-challenged muskmelon fruits, 19,224,475 and 22,962,561 methylated cytosines (mCs) were identified, respectively. Interestingly, pathogen infection led to a significant decrease (*p* < 0.05) in the overall proportion of methylated cytosines, with 59.46% in the control and 53.31% in the pathogen-challenged samples (Figure 4A, H=A, T or C).

A higher methylation level was observed in the CG context (83.49%/81.10%, the methylation levels of CG) compared to CHG (69.01%/62.43%, the methylation levels of CHG) and CHH (55.32%/48.75%, the methylation levels of CHH) contexts in the muskmelon fruits under both the control and pathogen challenge conditions (Figure 4A). In the control/pathogen-challenged samples, 14.60%/15.40% of overall cytosine methylation mCs were detected at CG sites, 10.20%/11.00% at CHG sites, and 75.10%/73.60% at CHH sites (Figure 4B). Analysis of the methylation levels between the control and pathogen-infected samples indicated an overall decrease in methylated cytosines in the gene bodies, promoters, and downstream regions. Among them, the promoters generally exhibited higher methylation levels compared to the gene bodies and downstream regions (Figure 4C). On the other hand, the pathogen infection caused a moderate fall in the methylation levels in the CG and CHG downstream areas and a broad decrease in the methylation levels in the CHH context, albeit to a lesser amount in different gene regions (Figure 4C).

### 3.4. Detection of Differentially Methylated Regions

**Figure 5:**
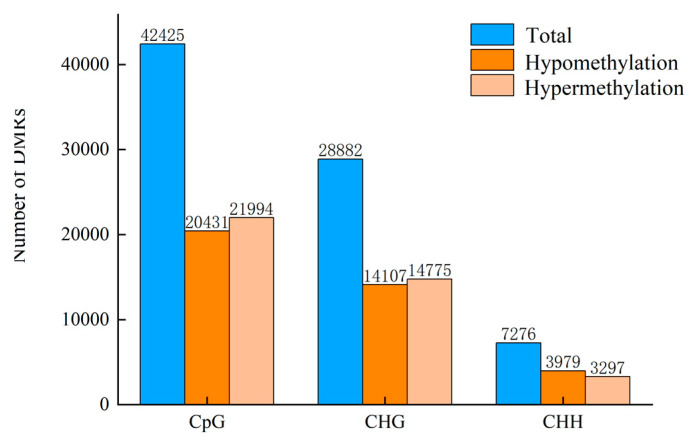
Total number of differentially methylated regions (DMRs) and hypermethylated and hypomethylated regions identified between control (CK) and pathogen-challenged (PC) fruits at 24 h post-infection.

The DMR quantity found in the pathogen-challenged muskmelon fruits (Figure 5) showed that although CG had the highest level of DNA methylation, there were 42,425 DMRs in CG, which was greater than in CHG and CHH. A total of 78,583 DMRs (40,066 hypermethylated and 38,517 hypomethylated) were observed in the pathogen-challenged samples compared to the control (Figure 5). The significant role of CG DNA methylation in response to the infection is indicated by the increased identification of DMRs in the CG context compared to the CHH and CHG contexts (Figure 5). To further characterize the identified DMRs, we categorized them into five groups: (I) DMRs in gene body regions, which included two subgroups of exon and intron DMRs; (II) DMRs in promoter regions; (III) DMRs in downstream regions; (IV) DMRs in intergenic regions (Table 1). For the CpG context, the most hyper- and hypo-DMRs were identified in Group I (19,044 hyper-DMRs and 18,053 hypo-DMRs) followed by Group IV (7219 and 6451), Group III (4809 and 4543), and Group II (4065 and 4165). Similar results were observed for the CHG and CHH contexts (Table 1). Although no statistically significant differences were observed in the number of hypermethylated and hypomethylated regions within CG DMRs, the levels of hypermethylation in the gene body and intergenic regions were relatively high. However, there were markedly higher levels of hypomethylation in the gene body regions of CHG and CHH (Table 1).

#### 3.4.1. Gene Ontology (GO) Enrichment of Genes Associated with DMRs

**Figure 6:**
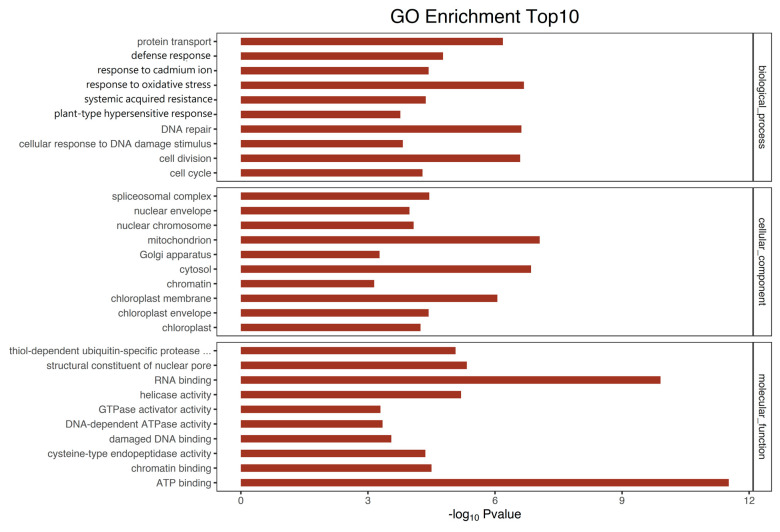
GO analysis of the DMR-associated genes. *p* values are corrected to –log10 (*p* values) ranging from 0 to infinity, and a lower *p* value (i.e., a greater –log_10_ (*p* value)) indicates a higher intensity. The top 10 GO enrichments of each categorization are listed in descending order of the *p*-value.

The GO enrichment analysis for the DMGs indicated that the three biological processes with the most significant changes included protein transport, defense response, and response to cadmium ions. Response to oxidative stress, systemic acquired resistance, plant-type hypersensitive response, DNA repair, cell division, and cellular responses to DNA damage stimuli were also notably affected (Figure 6). The primary molecular roles of the DMR-associated genes were RNA and ATP binding. The majority of the proteins linked to DMR were found in the nucleus, cytosol, mitochondrion, and chloroplast membrane (Figure 6).

#### 3.4.2. DMR-Associated Genes Involved in Early Defense Response During *T. roseum* Infection

During *T. roseum* infection, 176 DMGs were found in the *T. roseum*-inoculated muskmelon fruits at 24 hpi (Table S1), and some of them were involved in immune system processes and plant–pathogen interactions. Among these DMGs, 375 DMRs were identified, with 278 of them being hypomethylated, accounting for 74.13% of the total. The DMGs mainly encoded disease resistance family proteins, pathogen-related proteins, hypersensitive-induced response proteins, elicitor-responsive proteins, protein-enhanced disease resistance, glycine-rich proteins, and brassinosteroid LRR receptor kinases. Notably, 6 MLP-like proteins (MLP-like protein 28, 28 isoform X1, 43, 328, 329, and 423) and 19 DMGs responsible for coding MLO-like protein s were identified. It is worth noting that 4 DMGs (*MELO3C005504*, *MELO3C007539*, *MELO3C009806*, *MELO3C035030*) encoded disease resistance family proteins, *MELO3C018880* encoded glycine-rich protein, *MELO3C016546* encoded the phytohormone-binding protein-like family, and *MELO3C015872* encoded resistance to phytophthora 1, which were hypomethylated during incubation (Table S1). Additionally, some DMGs showed hypermethylation in response to biotic stimuli, such as *MELO3C005646* and *MELO3C018475* encoding elicitor-responsive protein 3 and DMGs (*MELO3C004385*, *MELO3C004386*, *MELO3C004387*) encoding a protein linked to pathogenicity PR-4-like protein.

Fifteen DMGs were involved in immune system processes (Table S1). Salicylic acid-binding protein 2, SNI1, metacaspase-1, NBS-LRR protein, RPM1-interacting protein 4, and other proteins were mostly encoded by these genes. Specifically, five DMGs (*MELO3C002777*, *MELO3C007531*, *MELO3C007532*, *MELO3C018425,* and *MELO3C021922*) encoding salicylic acid-binding protein 2 were hypomethylated in response to *T. roseum* infection. Another DMG (MELO3C005755) encoding nucleotide binding site–leucine-rich repeat protein was obviously hypomethylated during *T. roseum* infection under the CG context. Furthermore, the DMG (*MELO3C031111*) encoding SNI1 exhibited hypomethylation in the CG, CHG, and CHH contexts.

#### 3.4.3. DMGs Involved in ROS Metabolic and Response to Oxidative Process During *T. roseum* Infection

In this study, 134 DMGs related to ROS metabolic and response to oxidative processes attracted our attention. We found that 141 DMRs (72.30%) associated these DMGs were hypomethylated during incubation (Table S1). These genes primarily encoded peroxidase, catalase, ascorbate peroxidase, glutathione peroxidase, alpha-dioxygenase, aldehyde dehydrogenase, etc. Specifically, we identified 90 DMRs linked to 48 DMGs, encoding peroxidase genes, with 69 DMRs being hypomethylated and 21 DMRs being hypermethylated. These DMGs were found to be distributed across chromosomes 01–12 of *Cucumis melo* L. Notably, the gene *MELO3C017024*, which encodes catalase, exhibited hypomethylation. Furthermore, eight DMGs encoding ascorbate peroxidase and three DMGs encoding glutathione peroxidase were identified. Additionally, four DMGs (*MELO3C009933*, *MELO3C009931*, *MELO3C009933*, *MELO3C009931*) encoding alpha-dioxygenase 1, *MELO3C009932* encoding alpha-dioxygenase 1-like protein, and *MELO3C017346* encoding alpha-dioxygenase 2 were also found to be differentially methylated during incubation. *MELO3C013563* encoding metacaspase-5 was hypomethylated under the CHG context.

#### 3.4.4. DMGs Involved in Phenylpropanoid Metabolism During *T. roseum* Infection

A total of 41 DMGs related to phenylpropanoid metabolism and flavonoid biosynthetic processes were identified. These genes encode enzymes such as PAL, 4CL, chalcone–flavonone isomerase, leucoanthocyanidin dioxygenase, and caffeoylshikimate esterase. *MELO3C014229*, encoding the phenylalanine ammonia-lyase, was hypermethylated in exon regions under the CG context. Additionally, 5 DMGs (*MELO3C014222*, *MELO3C014223*, *MELO3C014228*, MELO3C017809, *MELO3C025786*) encoding phenylalanine ammonia-lyase-like enzymes, as well as 21 DMGs involved in encoding 4-coumarate CoA ligase and its analogues, were identified during incubation. Interestingly, *MELO3C025484*, encoding chalcone–flavonone isomerase, showed obvious hypomethylation, while a leucoanthocyanidin dioxygenase gene (*MELO3C030550*) was found to be hypermethylated in this study.

### 3.5. Association Between DNA Methylation and Gene Expression

In order to examine the changes in expression of DEGs, we performed RNA-sequencing (RNA-seq) analysis on muskmelon fruits collected at the identical developmental stages used in the WGBS. After removing low-quality reads, we acquired 50.65 million (CK) and 50.92 million (PC) raw reads for each respective group. The raw reads underwent further processing, resulting in over 46.14 million (CK) and 48.04 million (PC) uniquely mapped reads, which represented more than 91.08% and 91.34% of the total raw reads (Table S2). Of the uniquely mapped reads, the distributions were as follows: 87.53%/92.14% (CK/PC) aligned to exonic regions, 7.38%/4.78% to intronic regions, and 5.09%/3.08% to intergenic regions. In total, we identified 5487 DEGs (with a fold change ≥ 2 and an FDR ≤ 0.05), including 3671 that were upregulated and 1816 that were downregulated in the PC samples compared to the CK.

To systematically characterize the functional profiles of differentially expressed genes (DEGs), Gene Ontology (GO) term enrichment analysis (Q-value ≤ 0.05) was performed on muskmelon fruits infected with *T. roseum* (Table S3). Within the Biological Process category, the top three enriched terms were “cellular process” (GO:0009987), “metabolic process” (GO:0008152), and “response to stimulus” (GO:0050896). For molecular function, the most highly enriched terms were “binding” (GO:0005488), “catalytic activity” (GO:0003824), and “transporter activity” (GO:0005215). Notably, resistance-related GO terms were prominently enriched among upregulated genes, including “response to stimulus” (GO:0050896), “immune system process” (GO:0002376), “antioxidant activity” (GO:0016209), and “biological process involved in interspecies interaction” (GO:0044419), suggesting that *T. roseum* infection likely activates multi-layered defense mechanisms in the host (Table S3).

**Figure 7:**
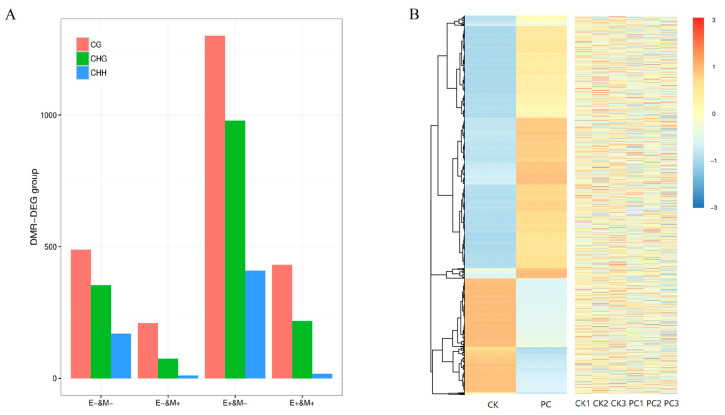
Representatives of differentially expressed genes with differential methylation in muskmelon fruits challenged with *T. roseum*. (**A**). The change trends of common DMGs and DEGs were statistically analyzed after the fruits were challenged with *T. roseum* (E+/E− indicates differentially upregulated/downregulated expression genes; M+/M− indicates differentially upregulated/downregulated methylated genes). (**B**). Heatmap displaying the abundance of DEGs that were differentially methylated and related with disease resistance in the CK (control) and PC (pathogen challenge) in the CG context. On the left, the expression of genes in different samples is represented by different colors. On the right, the methylation rate of DMR-related genes in different samples is represented by different colors. The red color indicates higher expression, and the blue color indicates lower expression.

Among the 5487 DEGs, 2899 in CG context, 662 in CHH context, and 1741 in CHG context DEGs harboring differential methylation were identified (Table S4). Notably, among the 5487 DEGs with differential methylation, 602 genes involved in defense responses were distributed as follows: 385 in the CG context, 43 in the CHH context, and 174 in the CHG context. Of the 602 defense-related genes, 439 (72.90%) were hypomethylated, while 163 were hypermethylated. Furthermore, 269 methylation sites were located in the gene body, 170 in the downstream 2 kb, and 163 in the upstream 2 kb. It can be found that the change in methylation degree under the CG context had the most obvious impact on the transcription of muskmelon fruits during the process of *T. roseum* infection, especially when hypomethylation occurred. There was a large number of genes with increased expression, followed by methylation in the CHG context, and the impact in the CHH context was the lowest (Figure 7).

**Figure 8:**
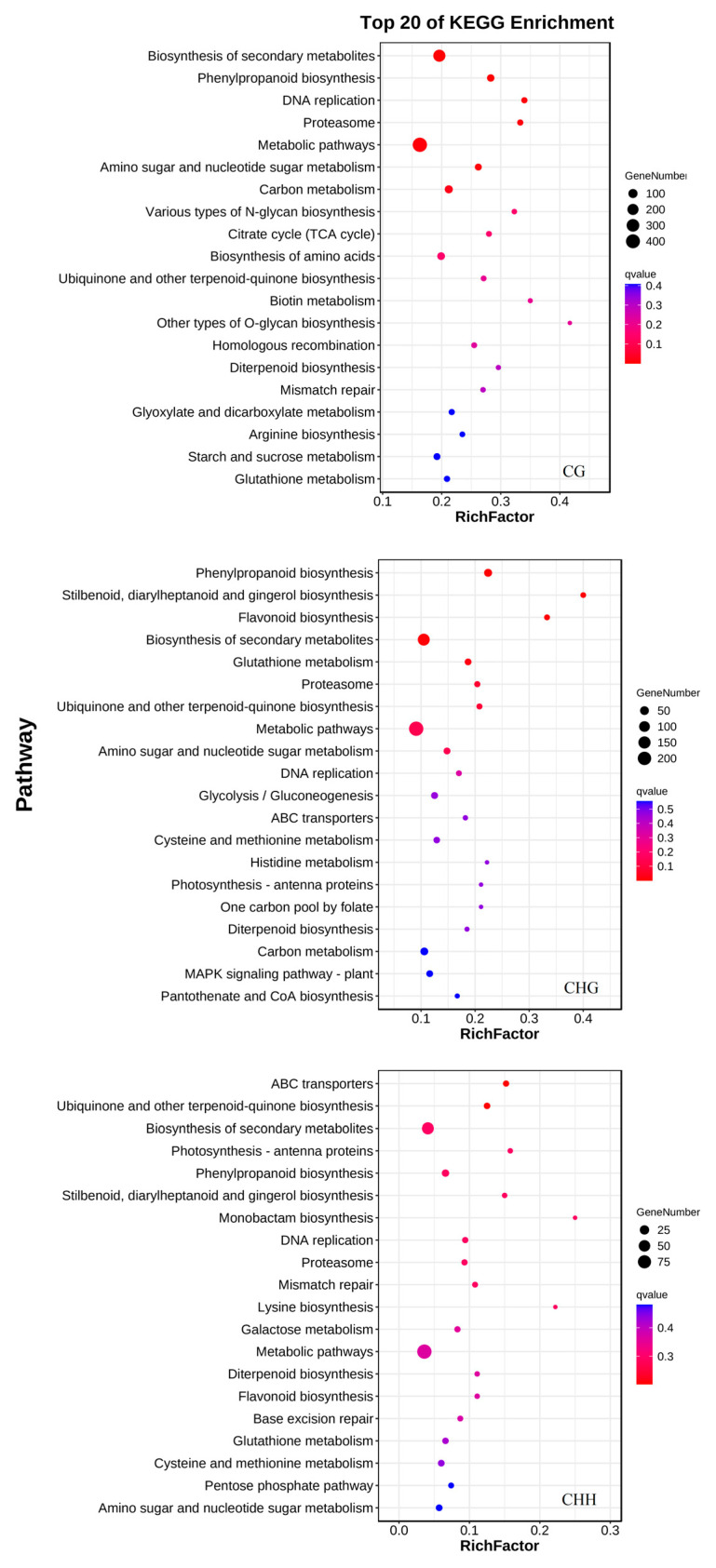
Statistics of the top 20 KEGG pathways enriched for the common genes of DMGs and DEGs in CG, CHG, and CHH contexts, respectively. The size of each circle represents the number of genes enriched in the corresponding pathway. The enrichment factor was calculated using the number of enriched genes divided by the total number of background genes in the corresponding pathway. A pathway with *p* < 0.05 was considered significantly enriched.

A total of 130, 101, and 122 KEGG pathways associated with the DEGs harboring differential methylation were discovered in the CG, CHH, and CHG contexts, respectively (Table S5). It is worth noting that phenylpropanoid biosynthesis (ko00940), biosynthesis of secondary metabolites (ko01110), DNA replication (ko03030), proteasome (ko03050), metabolic pathways (ko01100), ABC transporters (ko02010), and flavonoid biosynthesis (ko00941) were significantly enriched during *T. roseum* infection (Figure 8 and Table S5). We noticed that among the many DEGs harboring differential methylation related to phenylpropanoid biosynthesis, when the upstream 2kb of the genes were hypomethylated, the transcription levels were enhanced. For example, *MELO3C014652* encodes POD; its promoter region was hypomethylated by −12.78%, and its transcription was significantly elevated (log_2_(fold change, FC) = 5.83). *MELO3C023272* encodes cinnamyl alcohol dehydrogenase 9 (CAD9), and promoter hypomethylation was also accompanied by increased transcription. *MELO3C002421* encodes caffeoylshikimate esterase-like protein; upstream 2 kb hypomethylation by −25.40% was also accompanied by increased transcription (log_2_(FC) = 1.89). Of course, there are also some different situations, such *MELO3C022770*, encoding 4-coumarate-CoA ligase like 6 (4CLL6), and its upstream 2kb hypomethylation, while its transcript level decreased (log_2_(FC) = −2.16). *MELO3C003275* encodes peroxidase 47; its genebody was hypomethylated by −11.47%, and its transcription was increased (log_2_(FC) = 8.95).

## 4. Discussion

During the first 3 d of inoculation, no discernible symptoms were found in the two groups. However, fungal inoculation enhanced the ROS content in the muskmelons, with the peaks of H_2_O_2_ concentration at 24 hpi and O_2_^•−^ generation rate at 48 hpi in the tissues at the junction of decay and health regions of the muskmelons. These findings were consistent with research that has established the role of mitochondria in the production of ROS in the initial phases of fungal pathogen infection [21]. The swift and temporary generation of apoplastic ROS, referred to as an ‘oxidative burst’, is regarded as a vital component of the early response to both abiotic and biotic stressors in plants [36]. Xiao et al. found that *Ralstonia solanacearum* infection markedly elevated H_2_O_2_ content in eggplant roots at 12 hpi [37]. According to Huang et al. [38], ROS are essential for defense mechanisms against pathogens, plant hormone action, and programmed cell death. In plants, ROS often accumulate in response to abiotic stresses (like drought or UV radiation) or biotic stresses (like pathogen infection), which may lead to altered DNA methylation patterns and can affect gene expression and stress adaptation [39,40]. In order to inhibit pathogens development, it is hypothesized that infection-induced ROS signals within the first 24 hpi influence the redox state of the cell, modify the epigenetic regulation and expression of nuclear genes through retrograde regulation, and potentially initiate programmed cell death.

According to studies by Bi et al. [41] and Ge et al. [42], the phenylpropanoid pathway is known to significantly contribute to improving plant and fruit resistance to diseases. Our results indicated that the activities of PAL, C4H, 4CL, CAD, and POD were stimulated upon fungal pathogen infection, leading to an increase in lignin accumulation. This accumulation likely contributed to the production of sufficient metabolites in the fruit, creating physical barriers that hindered decay progression. Furthermore, Liu et al. indicated that the application of acibenzolar-S-methyl stimulated the phenylpropanoid pathway in muskmelon, leading to increased concentrations of antioxidants, including phenolics and flavonoids [19]. Hématy et al. highlighted that the biosynthesis of lignin in the cell wall strengthens it, serving as a physical defense against pathogen attacks and inhibiting the dissemination of pathogens [43]. The current study further identified the key nodes of the early defense response in the *T. roseum*-inoculated muskmelon at 24 hpi, highlighting the active involvement of ROS and phenylpropanoid metabolism in response to pathogens.

DNA methylation plays a crucial role in protecting host plants from pathogen invasion by modulating gene expression profiles, with its effects varying across different genomic regions and sequence contexts [44]. In our study, we observed a significant decrease of 6.15% in global DNA methylation levels in muskmelon fruits following *T. roseum* infection during the early defense response stage (Figure 4A). This finding aligns with previous research conducted in *Arabidopsis*, which demonstrated that reduced DNA methylation regulates pathogen-induced gene expression [45]. Furthermore, the methylation level in the CG context was the highest, followed by CHG, while CHH exhibited the lowest level (Figure 4), aligning with previous studies in plants such as rice [46] and *Arabidopsis* [47]. Notably, the overall methylation level in the control group was higher than that observed following pathogen challenge inoculation (Figure 4). The most abundant methylation sites were found in CHH, followed by CG, and the least abundant sites were found in CHG. This finding aligns with previous studies that shown the presence of methylation areas in the CHH sequence context as a result of *Cucumber Mosaic Virus* infections in tobacco [14] and *Cucumber Green Mottle Mosaic Virus* in watermelon [5]. The up-2k chromosomal region showed the highest levels of methylation in this study in the contexts of CG, CHG, and CHH (Figure 4C). Methylation of promoters (up-2k) can alter the structure of chromatin and prevent transcription from starting, which can affect gene expression [5]. Interestingly, the highest methylation levels in the CG context were observed within promoters, gene bodies, and downstream regions in both control and pathogen-challenged samples (Figure 4C). It is well established that methylation of gene bodies is linked to transcriptional activity and is essential for alternative splicing and the silencing of repetitive regions [48].

During *T. roseum* infection, the distribution of DMRs was mainly concentrated in the CG context, with hyper-DMRs being concentrated in exon and intron regions. On the other hand, within the CHH and CHG contexts, hypo-DMRs were localized within gene bodies. It is well established that methylation of gene bodies positively correlates with transcriptional activity and is essential for alternative splicing and the silencing of repetitive regions [48]. Remarkably, the gene promoter region of CG, CHG, and CHH all showed a trend of hypomethylation. Usually, there is an inverse relationship between promoter methylation and gene expression [49,50], suggesting that the early defense response triggered by pathogen infection may activate a greater number of gene responses.

Differential DNA methylation is essential for controlling the expression of genes linked to defense in response to harmful environmental factors or pathogen invasions [1]. In this study, 176 DMGs involved in the early stages of defense following *T. roseum* infection were shown to exhibit a tendency toward hypomethylation. Pathogen-related proteins have a role in the development of systemic acquired resistance against recurrent infections caused by bacteria, viruses, and fungi [2]. Specifically, four DMGs (*MELO3C008404*, *MELO3C016126*, *MELO3C016128*, and *MELO3C023694*) encoding pathogen-related proteins were found to be hypomethylated at their promoter regions and hypermethylated at their downstream regions. These DMGs are associated with defense responses and encode proteins such as disease resistance family protein, hypersensitive-induced response protein, and Protein ENHANCED DISEASE RESISTANCE 2. *Pseudomonas syringae* pv tomato DC3000 infection of *Arabidopsis* causes hypomethylation in genomic areas linked to genes involved in plant defense [51]. The interaction between plants and pathogens affects the communication between key signaling molecules and pathways involved in innate immune systems, such as jasmonic acid (JA), salicylic acid (SA), abscisic acid (ABA), auxin IAA, and brassinosteroid BR [52,53,54]. In our study, five DMGs encoding salicylic acid-binding protein 2, *MELO3C025152* encoding coronatine-insensitive 1 (receptors in the jasmine signaling pathway), *MELO3C026838* encoding brassinosteroid LRR receptor kinase, and *MELO3C031325* encoding NBS-LRR protein were simultaneously hypomethylated during *T. roseum* infection. These findings are consistent with research indicating that DNA methylation may facilitate plant hormone-mediated signal transmission during fungal infection [55].

ROS homeostasis mediated by DNA methylation plays an important role in plant development and responses to a variety of stressors [56,57,58]. ROS not only cause direct oxidative damage but also influence epigenetic marks like DNA methylation in the context of immune responses [40]. A total of 134 DMGs were enriched in the ROS metabolic pathway, with a majority encoding antioxidant enzymes, including POD, CAT, glutathione peroxidase (GSH-Px), and ascorbate peroxidase (APX) in this study. A large number of DMGs encoding peroxidase genes showed hypomethylated modifications, aligning with the increase of POD activity at 24 hpi. It can be seen that the strong oxygen burst during muskmelon-*T. roseum* interactions could influence the ability to scavenge ROS through DNA methylation processes.

The phenylpropanoid pathway serves as a crucial source of precursors for various secondary metabolites in plants, such as lignin, flavonoids [19]. PAL acts as the primary enzyme in this pathway, initiating the biosynthesis of cinnamic acid and serving as a precursor for other phenolic compounds [59]. *MELO3C014223*, which encodes a phenylalanine ammonia-lyase-like enzyme, exhibited hypomethylation in its promoter regions, potentially reducing the repressive impact of DNA methylation on adjacent genes. In total, 21 differentially methylated genes (DMGs) were identified, including those responsible for encoding 4-coumarate CoA ligase and related enzymes, which are involved in regulating the flow of various phenylpropanoid biosynthetic pathways during pathogen attacks. Moreover, it has been demonstrated that flavones function as phytoalexins, contributing to plant defense mechanisms [60]. In this study, chalcone–flavonone isomerase involving the flavonoid biosynthetic process was identified in muskmelon during *T. roseum* infection. Defense reactions alter CpG DNA methylation levels and increase the synthesis of stress-related secondary metabolites inside *Lactuca sativa* [61]. Three DMGs (*MELO3C002571*, *MELO3C002421, MELO3C017954*) encoding caffeoyl shikimate esterase (CSE) are involved in the lignin biosynthesis pathway [62] and exhibit hypomethylation in their promoter regions. The findings imply that *T. roseum* infection, at least in part, through the demethylation of relevant gene promoters, increases disease resistance and the phenylpropanoid pathway, along with associated secondary metabolites.

RNA-seq analysis of muskmelon fruits at developmental stages corresponding to the WGBS data detected 5487 differentially expressed genes (DEGs), with 3671 upregulated and 1816 downregulated in the inoculated samples (PC) compared to the controls (CK). These results revealed substantial transcriptomic alterations following *T. roseum* inoculation, which were consistent with research on powdery mildew resistance mechanisms in melon [63]. Notably, upregulated genes were significantly enriched in resistance-related functional categories, including “response to stimulus”, “immune system process”, “antioxidant activity”, and “biological processes involved in interspecies interaction”, indicating systemic activation of defense mechanisms. These findings align with our previous proteomic study [20], which demonstrated that *T. roseum* infection in muskmelon fruits reprograms energy metabolism and induces ROS accumulation. The observed transcriptional changes may be linked to post-inoculation DNA hypomethylation, particularly in gene promoter regions. Demethylation is known to enhance transcriptional accessibility [45], and our data support this mechanism (Table S2): hypomethylation of genes associated with ROS metabolism and the phenylpropanoid biosynthesis pathway coincided with elevated expression of antioxidant enzymes (e.g., peroxidases) and lignin synthesis-related genes (e.g., PAL, 4CL).

Methylation variations in genomic regions frequently correlate with changes in gene expression at the transcriptional stage across a range of organisms [64]. In this study, 2899 genes in the CG context, 662 genes in the CHH context and 1741 genes in the CHG context out of the DMGs displayed distinct expression levels in muskmelons during the initial defense response to *T. roseum* infection (Table S3). Notably, 2899 differentially expressed genes harboring differential methylation were concentrated in the CG context, with the majority being upregulated due to hypomethylation (E+ and M–, Figure 7A). In *Arabidopsis*, the role of reduced DNA methylation is crucial for the regulation of gene expression induced by pathogens in both hypomethylated and hypermethylated mutant strains [45]. In plants, an association has been established between the methylation within gene bodies in the mCG context and enhanced gene expression, especially concerning constitutive gene expression [65]. After analyzing the top 20 KEGG pathways enriched by the differentially expressed genes harboring differential methylation, it was observed that the enriched pathways, in the context of CG, CHG, and CHH, exhibited similarities to some extent (Figure 8). It is worth noting that the enriched KEGG pathways include pathways of biosynthesis of secondary metabolites and phenylpropanoid biosynthesis, especially some genes encoding key enzymes in phenylpropanoid metabolism, including the genes encoding CAD, POD, 4CL and their analogues, which were transcriptionally upregulated. These results were consistent with our biochemical results. Among them, *MELO3C014652* encoding POD, *MELO3C023272* encoding CAD9, and *MELO3C002421* encoding caffeoylshikimate esterase-like protein were hypomethylated in their promoter regions, and transcription was upregulated. Melatonin enhances gene expression through the reduction of promoter methylation, consequently facilitating disease resistance and the biosynthesis of flavonoids [66]. Overall, these results indicate that the intricacies of DNA methylation and its influence on gene expression regulation in muskmelon fruits are complex issues that necessitate additional exploration.

## 5. Conclusions

DNA methylation of muskmelon fruits decreased globally after *T. roseum* inoculation. There were more DMRs and a greater degree of methylation at CG sites. Reduced promoter methylation has been linked to changes in a number of DMGs that are related to defense response, metabolism of ROS, and phenylpropanoid metabolism. Furthermore, muskmelon fruits showed enhanced ROS accumulation, increased enzymes activity involved in the phenylpropanoid pathway, and accelerated lignin accumulation at 24 hpi. The analysis of the relationship between gene expression and DMGs indicates that DEGs with differential methylation were primarily influenced by hypomethylation, which may explain the observed increase in related transcript levels. These results will aid in comprehending the molecular mechanisms underlying the early defense reactions of postharvest fruits against fungal infections at the methylation level.

## Figures and Tables

**Table 1:** DMRs identified in muskmelon at 24 hpi (CK, control check; PC, pathogen challenge).

PC vs. CK 24 h Comparison	Total DMRs	Hyper DMRs	Hypo DMRs	Exon Hyper	Exon Hypo	Intron Hyper	Intron Hypo	Promoter Hyper	Promoter Hypo	Downstream Hyper	Downstream Hypo	Intergenic Hyper	Intergenic Hypo
**CpG**	42,425	21,994	20,431	9359	8973	9685	9080	4065	4165	4809	4543	7219	6451
**CHG**	28,882	14,775	14,107	1809	2076	2342	2907	2539	2617	1889	1858	8297	7383
**CHH**	7276	3297	3979	516	788	442	656	903	1068	608	651	1499	1802

## Data Availability

The original contributions presented in this study are included in the article/Supplementary Materials. Further inquiries can be directed to the corresponding author.

## Supplementary Materials

The following supporting information can be downloaded at: https://www.mdpi.com/article/10.3390/jof11040243/s1, Table S1: Data of differentially methylated regions (DMRs); Table S2: Summary of the RNA-Seq data; Table S3 GO term analysis of DEGs; Table S4: The subset of DMR-associated genes with differentially expressed genes (DEGs); Table S5: KEGG pathways of the subset of genes that are differentially expressed in DMGs.
